# Effect of synbiotic bread containing lactic acid on glycemic indicators, biomarkers of antioxidant status and inflammation in patients with type 2 diabetes: a randomized controlled trial

**DOI:** 10.1186/s13098-019-0496-9

**Published:** 2019-12-05

**Authors:** Atie Ghafouri, Mitra Zarrati, Farzad Shidfar, Iraj Heydari, Raheleh Shokouhi Shoormasti, Omid Eslami

**Affiliations:** 1 Department of Nutrition, School of Public Health, Iran University of Medical Sciences, Tehran, Iran; 2 Institute of Endocrinology and Metabolism, Iran University of Medical Sciences, Tehran, Iran; 3 Immunology, Asthma and Allergy Research Institute, Tehran University of Medical Sciences, Tehran, Iran

**Keywords:** Synbiotic, Lactic acid, Glycated hemoglobin A, Oxidative stress, Diabetes

## Abstract

**Background:**

The aim of this randomized, double-blinded, controlled trial was to investigate the effect of daily consumption of a synbiotic bread containing lactic acid on glycemic status, antioxidant biomarkers and inflammation in patients with type 2 diabetes (T2D).

**Methods:**

T2D patients, aged 20 to 60 years, were randomly assigned to consume synbiotic + lactic acid (n = 30), synbiotic (n = 30), lactic acid (n = 30), or control (n = 30) bread for 8 weeks. Patients consumed bread 3 times a day in a 40 g package for a total of 120 g/day. Glycemic status, antioxidant capacity, and serum hs-CRP were assessed before and after the intervention.

**Results:**

Of a total of 120 patients that were included in the study, 100 completed the trial. In the adjusted analysis, it was found that consumption of synbiotic + lactic acid bread caused a significant decrease in HbA1c compared to the control bread (− 0.41 ± 0.33 vs 0.004 ± 0.10%, respectively; *P *< 0.001), while it significantly increased serum superoxide dismutase (SOD) (0.87 ± 1.14 vs. 0.18 ± 0.85 mmol/L, *P *= 0.02). Also, changes in glutathione peroxidase (GSH-Px) were significantly higher following the consumption of synbiotic + lactic acid bread than lactic acid bread. However, it had no significant effect on fasting plasma glucose, serum insulin, and total antioxidant capacity.

**Conclusion:**

Overall, daily consumption of a synbiotic bread containing lactic acid for 8 weeks had beneficial effects on HbA1c, SOD, and GSH-Px among T2D patients.

*Trial registration* This study was registered in Iranian Registry of Clinical Trials with number: IRCT201505242709N33 (Registration date: 2015-11-23, http://www.irct.ir/trial/2544)

## Background

Type 2 diabetes (T2D) is a common metabolic disorder in which the prevalence rate is increasing all over the world, estimating to reach 552 million by 2030 [1]. It is characterized by insulin resistance, insulin deficiency, and declined pancreatic beta-cell function [2]. Also, there is accumulating evidence that suggests the potential role of oxidative stress and inflammation in the development of T2D and its complications [3, 4]. If the disease remained uncontrolled, it could be accompanied by various adverse outcomes, including cardiovascular disease, renal failure, neuropathy, and diabetic retinopathy [5]. Despite the presence of many pharmacological agents, diet remains an essential aspect of the overall management of T2D [6].

In recent years, great attention has been paid to the role of synbiotic foods, which is a combination of both probiotics and prebiotics, on the metabolic health of T2D patients. Several clinical trials reported that synbiotics foods have a favorable effect on insulin resistance, inflammation, and oxidative stress [7–10]. The beneficial effects of synbiotic on glucose homeostasis might be the result of the production of short-chain fatty acids (SCFAs), which could increase glucagon-like peptide-1 (GLP-1) secretion, an incretin hormone that decreases plasma glucose, improve insulin secretion, and regulate pancreatic beta-cell function [11]. Also, they could improve the composition of gut microbiota and intestinal integrity as well as suppress the production of proinflammatory mediators such as interleukin-6 (IL-6) and tumor necrosis factor-α (TNF-α), and therefore may reduce inflammation, oxidative stress and insulin resistance [11, 12].

The efficacy of low-glycemic index (GI) foods on the glycemic status of T2D patients has been extensively investigated in the literature. The results of a recent meta-analysis of clinical trials showed a significantly higher reduction in glycated hemoglobin A (HbA1c) and fasting plasma glucose (FPG) following consumption of low-GI diets compared to the high-GI or control diets in T2D patients [13]. Thus, modulating the GI features of carbohydrate-rich sources may be an effective strategy in the management of glucose metabolism in diabetic patients. It is hypothesized that adding lactic acid to carbohydrate-rich foods could decrease the rate of delivery of glucose to the blood. Several studies in experimental models or healthy subjects showed the favorable effect of a bread-containing lactic acid on improving glucose homeostasis and insulin responses [14–16], which this might be due to the reduction in the rate of amylolysis, a process in which the starch converts into sugar in bread [16].

Current evidence regarding the efficacy of synbiotic foods in patients with T2DM is limited, and also, most of the clinical trials that used synbiotic or lactic acid bread have been conducted in the non-diabetic population. To our knowledge, no study has examined the effect of consumption of a synbiotic bread containing lactic acid on the metabolic profile of T2D patients. Therefore, the present clinical trial aimed to investigate the effect of consumption of a synbiotic bread with lactic acid on glycemic status, inflammation, and antioxidant capacity in patients with T2D. We hypothesized that consumption of a synbiotic bread with added lactic acid may exert synergic effect and could contribute to a greater improvement in metabolic parameters of diabetic patients rather than consumption of synbiotic or lactic acid bread alone.

## Methods

### Participants

This randomized, double-blinded, controlled clinical trial was conducted in Tehran, Iran, between March to December 2016. Subjects were recruited from Firouzgar Clinic of Endocrinology and Metabolism affiliated to Iran University of Medical Sciences. Based on the criteria of American Diabetes Association, subjects were considered to have T2D if they had one of the following items: FPG ≥ 126 mg/dL; Post Prandial (2-h) glucose ≥ 200 mg/dL; and HbA1c ≥ 6.5% [2]. Exclusion criteria were: age below than 20 and higher than 60 years old, taking any medication except for glucose-lowering drugs, using insulin, pregnancy, lactation, and having metabolic, cardiovascular, renal or thyroid diseases as well as cancer or allergy.

We estimated the required sample size based on the differences in serum insulin obtained from a similar study [10]. Considering the type 1 error 5% (α = 0.05) and type 2 error 20% (β = 0.2; power = 80%), 25 subjects in each group was required, and therefore, we included 100 subjects in this trial. The Ethics Committee of Iran University of Medical Science confirmed the study protocol (Code no. IR.IUMS.REC.1394.26524) and written informed consent was gathered from all participants. Also, this study was registered in the Iranian Registry of Clinical Trials (IRCT no. IRCT201505242709N33).

### Study design

Before the beginning of the intervention, all participants entered into a 2 week run-in period, in which they had to avoid consumption of any other food or supplements containing synbiotic or lactic acid. At the end of the run-in period, subjects were randomly allocated into four equal groups using balanced block randomization method with matched subjects in each block based on sex and body mass index (BMI < 30 kg/m^2^ and BMI ≥ 30 kg/m^2^).

Participants were assigned to receive either one of the following bread daily for 8 weeks including (1) control bread containing beta-glucan (3 g); (2) lactic acid bread containing beta-glucan (3 g) and lactic acid (4 g); (3) synbiotic bread containing beta-glucan (3 g), *Bacillus coagulans* (1×10^8^ CFU), and inulin (10 g); and (4) synbiotic + lactic acid bread containing beta-glucan (3 g), *Bacillus coagulans* (1×10^8^ CFU), inulin (10 g), and lactic acid (4 g). Participants were asked to consume the bread in a 40-g package 3 times a day.

All types of bread were provided by the Forni Bread Company, Tehran, Iran. Inulin was extracted from oat bran (Iran Tejarat Company), and beta-glucan was extracted from barley. *Bacillus coagulans* was provided by Zist Takhmir Pharmaceutical Company (Tehran, Iran). The nutritional composition of the study bread per 100 g was as follows: energy, 260 kcal; carbohydrate, 47.2 g; protein, 9.2 g; fat, 4 g; moisture, 37.2; and ash 2.4. They were packed in identical packaging and were labeled as A, B, C, and D by the producer, so the study participants, dietitian, laboratory staff and data analysis specialist were blinded to the type of intervention. Participants received a 1-week supply of their bread every week.

They were advised to maintain their usual diet and physical activity, and not to consume any bread other than that provided to them during the intervention. Patients’ compliance with the consumption of bread was monitored by telephone interviews once a week. Dietary intake of participants was recorded using 24-h dietary recalls for 3 nonconsecutive days at the beginning and after the intervention. The average intake of energy, macro- and micro-nutrients were analyzed using Nutritionist IV software (First Data Bank; Hearst Corp, San Bruno, CA, USA).

### Assessment of anthropometric measures

Anthropometric measurements including weight, and height were obtained at baseline and the end of the intervention. Body weight was measured by a digital scale (Seca, Hamburg, Germany) to the nearest 0.1 kg, in an overnight fasted state without shoes and in minimal clothing. Height was measured by a stadiometer (Seca, Hamburg, Germany) to the nearest 0.5 cm. BMI was calculated as weight/height^2^ (kg/m^2^).

### Clinical laboratory assessment

Blood samples (10 cc) were taken at the baseline and at the end of study at Firouzgar laboratory of Endocrine and Metabolism Centre after an overnight fast. FPG was assayed using a standard enzymatic method with commercial kits (Pars Azmun, Tehran, Iran). HbA1c levels were measured by DS5 device and ESCALON Reagent (REF:14-35) kit. Serum insulin levels were assayed by ELISA method (Beckman Coulter-France). The homeostasis model of assessment-insulin resistance (HOMA-IR) was determined according to the standard formula [10]. Serum high sensitivity C-reactive protein (hs-CRP) levels were assessed by the immunoturbidometery method with commercial kits (Pars Azmun, Tehran, Iran). Serum levels of total antioxidant capacity (TAC), superoxide dismutase (SOD) and glutathione peroxidase (GSH-Px) were assayed by ELISA method (ZellBio GmbH, Germany).

### Statistical analysis

All statistical analyses were performed using SPSS software version 22 (SPSS Inc., Chicago, IL, USA). Kolmogorov–Smirnov test was used to check the normality of the quantitative variables. Data with normal distribution were reported as mean and standard error of the mean (SEM), while those with non-normal distribution were represented as median and interquartile ranges. To compare between-group differences, one-way ANOVA or Kruskal–Wallis test were performed. In case of any significant difference between the study groups, post hoc tests were performed. Also, analysis of covariance (ANCOVA) was used adjusting for age, weight, BMI, and baseline values of variables. Results with *P*-value < 0.05 were considered statistically significant.

## Results

**Fig. 1:**
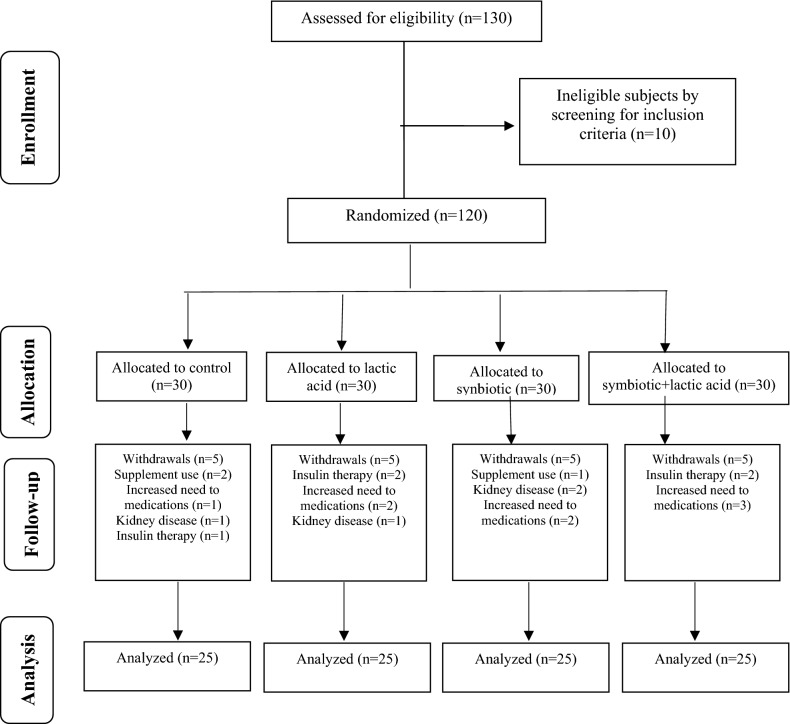
Flow diagram of the trial

Of a total of 130 subjects that enrolled initially in the study, 120 were eligible for participation and were randomly allocated to the study groups. During the intervention, 20 subjects were excluded due to the following reasons: using supplements, increased the need for medications, kidney disease, and insulin therapy. Finally, 100 participants (57 males and 43 females) completed the study (Fig. 1. Patient flow diagram). No serious side effects were reported following the consumption of bread.

Table 1 shows the general characteristics of the study participants. There were no significant differences in terms of age, anthropometric measures and medication use between the study groups. Also, none of the patients had changed their medications during the 8-weeks of intervention. Moreover, the 3-day averages of energy, macro- and micro-nutrient intakes were not significantly different between the study groups at the beginning and after the intervention (Table 2).

**Table 1:** General characteristic of study participants

Variable	Study groups				*P*-value ^a^
	I: Control bread ( n = 25)	II: Lactic acid bread (n = 25)	III: Synbiotic bread (n = 25)	IV: Synbioti + lactic acid bread (n = 25)	
Age (years)	54.60 ± 0.83	55.00 ± 0.97	54.92 ± 1.02	53.88 ± 1.09	0.84
Height (cm)	168.48 ± 1.57	169.60 ± 1.59	170.96 ± 1.47	167.72 ± 1.53	0.47
Weight at study baseline (kg)	75.80 ± 0.93	75.48 ± 1.67	76.28 ± 0.79	74.64 ± 1.26	0.80
Weight at end-of-trial (kg)	75.72 ± 0.90	75.48 ± 1.59	75.76 ± 0.79	74.72 ± 1.23	0.91
Weight change (kg)	− 0.08 ± 0.32	0.00 ± 0.29	− 0.52 ± 0.21	0.08 ± 0.34	0.67
BMI at study baseline (kg/m^2^)	27.04 ± 0.50	26.33 ± 0.46	26.39 ± 0.51	26.83 ± 0.42	0.67
BMI at end-of-trial (kg/m^2^)	26.61 ± 0.41	26.28 ± 0.44	25.70 ± 0.50	26.30 ± 0.41	0.54
BMI change (kg/m^2^)	− 0.44 ± 0.19	− 0.05 ± 0.14	− 0.68 ± 0.24	− 0.52 ± 0.18	0.14
Metformin use (n/day)	2.00 ± 0.12	2.00 ± 0.28	1.70 ± 0.26	2.00 ± 0.22	0.98
Glibenclamide use (n/day)	1.2 ± 0.16	1.00 ± 0.22	2.00 ± 0.18	1.80 ± 0.22	0.88

All values are mean ± SEM

*BMI* body mass index

^a^Obtained from One-way ANOVA

**Table 2:** Dietary intake of study participants after 8 weeks of intervention

Variable	Study groups	Baseline	End-of-trial	*P*-value^a^
Energy (kcal/day)	I: Control bread	2203.21 ± 81.02	2192.00 ± 70.79	0.87
	II: Lactic acid bread	2094.62 ± 58.68	2130.44 ± 59.14	
	III: Synbiotic bread	2212.38 ± 81.17	2138.17 ± 56.97	
	IV: Synbiotic + lactic acid bread	2085.71± 57.79	2130.28 ± 58.66	
Carbohydrate (g/day)	I: Control bread	313.44 ± 8.61	311.08 ± 8.55	0.22
	II: Lactic acid bread	295.04 ± 8.91	295.72 ± 7.00	
	III: Synbiotic bread	313.44 ± 8.61	311.68 ± 8.44	
	IV: Synbiotic + lactic acid bread	295.04 ± 8.91	293.44 ± 7.88	
Protein (g/day)	I: Control bread	70.64 ± 6.50	69.84 ± 5.62	0.14
	II: Lactic acid bread	59.60 ± 3.73	57.00 ± 3.13	
	III: Synbiotic bread	70.64 ± 6.50	69.48 ± 5.63	
	IV: Synbiotic + lactic acid bread	59.60 ± 3.73	60.52 ± 4.26	
Total fat (g/day)	I: Control bread	81.84 ± 3.36	80.48 ± 2.84	0.84
	II: Lactic acid bread	79.72 ± 2.09	78.28 ± 2.41	
	III: Synbiotic bread	82.16 ± 3.19	79.44 ± 2.62	
	IV: Synbiotic + lactic acid bread	79.70 ± 2.09	77.23 ± 13.79	
SFA (g/day)	I: Control bread	22.47 ± 1.30	20.62 ± 0.95	0.24
	II: Lactic acid bread	18.99 ± 0.55	19.75 ± 0.93	
	III: Synbiotic bread	22.47 ± 1.30	22.84 ± 1.22	
	IV: Synbiotic + lactic acid bread	18.99 ± 0.55	21.26 ± 1.21	
TDF (g/day)	I: Control bread	14.27 ± 0.53	14.58 ± 0.47	0.75
	II: Lactic acid bread	15.14 ± 0.67	14.60 ± 0.75	
	III: Synbiotic bread	13.32 ± 0.60	14.19 ± 0.49	
	IV: Synbiotic + lactic acid bread	15.10 ± 0.63	15.06 ± 0.50	
Vitamin D (μg/day)	I: Control bread	5.07± 0.12	5.02 ±0.11	0.44
	II: Lactic acid bread	4.75 ± 0.14	4.95 ± 0.14	
	III: Synbiotic bread	5.07 ± 0.12	4.79 ± 0.14	
	IV: Synbiotic + lactic acid bread	4.75 ± 0.14	4.76 ± 0.12	
Vitamin C (mg/day)	I: Control bread	17.28 ± 0.72	16.61 ± 0.61	0.85
	II: Lactic acid bread	16.88 ± 0.59	16.97 ± 0.58	
	III: Synbiotic bread	17.28 ± 0.72	17.15 ± 0.69	
	IV: Synbiotic + lactic acid bread	16.88 ± 0.59	17.36 ± 0.55	
Zinc (mg/day)	I: Control bread	7.38 ± 0.21	7.16 ± 0.21	0.18
	II: Lactic acid bread	5.72 ± 0.20	6.52 ± 0.27	
	III: Synbiotic bread	7.38 ± 0.21	6.84 ± 0.21	
	IV: Synbiotic + lactic acid bread	5.72 ± 0.20	6.58 ± 0.20	

All values are mean ± SEM

*SFA* saturated fatty acid, *TDF* total dietary fiber

^a^Obtained from One-way ANOVA

Table 3 compares the changes in glycemic measures, antioxidant capacity, and hs-CRP between the study groups after 8 weeks of intervention. At the end of the trial, FPG,HbA1c, and hs-CRP decreased significantly compared to the baseline (*P *= 0.01, *P *< 0.001, and *P *= 0.01, respectively) in the synbiotic +lactic acid bread group, while SOD and GSH-Px significantly increased (*P *= 0.001 and *P *= 0.002, respectively). Also, synbiotic bread group showed a significant reduction in HbA1c (*P* < 0.001) after 8 weeks of intervention, although SOD and GSH-Px increased significantly (*P *= 0.006 and *P *= 0.04, respectively). Moreover, HbA1c and serum insulin had a significant decrease in lactic acid bread group at the end of the intervention (*P *= 0.01, and *P* = 0.004, respectively). Furthermore, those consuming control bread containing beta-glucan showed a significant reduction in serum insulin and HOMA-IR after 8 weeks of trial (*P *< 0.001 and *P* = 0.02, respectively).

**Table 3:** Comparison of biochemical measures between the study groups at baseline and at the end of the intervention

Variable	Study groups	Baseline	End-of-trial	Change	*P*-value^a^	*P*-value^b^	*P*-value^c^
FPG (mg/dL)	I: Control bread	122.56 ± 5.87	120.88 ± 4.47	− 1.68 ± 6.43	0.61	0.54	0.6
	II: Lactic acid bread	127.48 ± 4.95	115.16 ± 3.81	− 12.32 ± 6.29			
	III: Synbiotic bread	126.84 ± 6.08	120.80 ± 4.43	− 6.04 ± 8.41			
	IV: Synbiotic+ lactic acid bread	129.68 ± 4.88	115.60 ± 3.55	− 14.08 ± 5.56			
HbA1c (%)	I: Control bread	6.98 ± 0.14	6.99 ± 0.14	0.004 ± 0.02	0.005^*^	<0.001**	<0.001***
	II: Lactic acid bread	6.84 ± 0.13	6.69 ± 0.11	− 0.14 ± 0.05			
	III: Synbiotic bread	6.78 ± 0.11	6.50 ± 0.08	− 0.28 ± 0.06			
	IV: Synbiotic + lactic acid bread	6.84 ± 0.12	6.43 ± 0.11	− 0.41 ± 0.06			
HOMA-IR	I: Control bread	2.12 ± 0.27	1.81 ± 0.24	− 0.30 ± 0.12	0.31	0.11	0.09
	II: Lactic acid bread	2.12 ± 0.25	2.27 ± 0.22	0.14 ± 0.10			
	III: Synbiotic bread	2.32 ± 0.24	2.35 ± 0.22	0.03 ± 0.07			
	IV: Synbiotic + lactic acid bread	2.20 ± 0.21	2.23 ± 0.21	0.03 ± 0.09			
Insulin (µU/mL)	I: Control bread	8.18 ± 0.95	6.68 ± 0.81	− 1.51 ± 0.31	0.96	0.91	0.96
	II: Lactic acid bread	7.60 ± 0.73	6.31 ± 0.62	− 1.28 ± 0.39			
	III: Synbiotic bread	8.25 ± 0.95	6.19 ± 0.83	− 2.05 ± 1.03			
	IV: Synbiotic + lactic acid bread	7.73 ± 0.73	6.23 ± 0.65	− 1.50 ± 1.08			
TAC (mmol/L)	I: Control bread	0.38 ± 0.01	0.37 ± 0.01	− 0.01 ± 0.01	0.27	0.11	0.26
	II: Lactic acid bread	0.37 ± 0.01	0.35 ± 0.01	0.02 ± 0.02			
	III: Synbiotic bread	0.38 ± 0.01	0.38 ± 0.007	− 0.007 ± 0.01			
	IV: Synbiotic+ lactic acid bread	0.35 ± 0.01	0.38 ± 0.01	0.03 ± 0.01			
SOD (mmol/L)	I: Control bread	12.41 ± 0.43	12.23 ± 0.40	0.18 ± 0.17	<0.001^‡^	0.001^‡‡^	<0.001^‡‡‡^
	II: Lactic acid bread	17.75 ± 0.35	17.21 ± 0.48	− 0.54 ± 0.40			
	III: Synbiotic bread	22.09 ± 0.71	22.49 ± 0.67	0.40 ± 0.13			
	IV: Synbiotic+ lactic acid bread	18.87 ± 0.92	19.74 ± 0.91	0.87 ± 0.22			
GSH-Px (µmol/L)	I: Control bread	44.37 (26.62, 66.56)	44.9 (26.90, 66.80)	0.47 – (0.91, 2.89)	0.30^d^	0.48^d^	0.04^†^
	II: Lactic acid bread	70.88 (39.93, 173)	51 (36.25, 118.5)	1.23 (− 29.55, 7.10)			
	III: Synbiotic bread	115.38 (31.06, 244)	123 (31.06, 245.5)	0.85 (0.000, 1.54)			
	IV: Synbiotic+ lactic acid bread	54.38 (31.06, 186)	72 (38.25, 209)	1.19 (0.40, 2.86)			
hs-CRP (mg/L)	I: Control bread	699 (391, 2499)	580 (395, 1063)	519.35 ± 304.35	0.7	0.48	0.02^††^
	II: Lactic acid bread	926 (501, 1782)	749 (522, 1573)	33.80 ± 237.60			
	III: Synbiotic bread	840 (528, 1341)	687 (454, 960)	− 689.76 ± 368.98			
	IV: Synbiotic+ lactic acid bread	926 (488, 1887)	750 (487, 1063)	− 575.96 ± 268.60			

Values are presented as mean ± SEM or median (Q1, Q3)

*FPG* fasting plasma glucose, *HOMA-IR* homeostatic model assessment of insulin resistance, *TAC* total antioxidant capacity, *GSH-Px* glutathion peroxidas, *hs-CRP* high sensitivity C-reactive protein

^a^*P*-values represent the differences between the study groups after 8 weeks of intervention obtained from One-way ANOVA or Kruskal**–**Walis test

^b^*P*-values represent the changes in metabolic parameters between the study groups obtained from One-way ANOVA Kruskal–Walis test

^c^*P*-value represent the changes in metabolic parameters between the study groups obtained from ANCOVA adjusted based on age, weight, BMI and baseline values of variables

^d^*P*-values obtained from Kruskal–Walis test

* Significant differences between groups I and IV (*P *= 0.006) and Groups I and III (*P *= 0.02)

** Groups I and III (P = 0.002), I and IV (*P *< 0.001), and II and IV (*P *= 0.004)

*** Groups I and III (*P *< 0.001); I and IV (*P *< 0.001), and II and IV (*P* = 0.001)

^‡^Significant differences between Groups I and II (*P *< 0.001), I and III (*P *< 0.001), and I and IV(*P *< 0.001)

^‡‡^Groups I and IV (*P *= 0.02) and II and IV (*P *= 0.01)

^‡‡‡^Groups I and IV (*P *= 0.005), II and III (P = 0.01), and II and IV (*P *= 0.001)

^†^Significant difference between Groups II and IV (*P *= 0.03)

^††^Significant difference between Groups II and III (*P *= *0.02*)

The baseline values of variables were not significantly different between the study groups (*P*-values are not shown). Serum HbA1c was significantly lower in the synbiotic + lactic acid bread group and also, synbiotic bread group compared to the control group at the end of trial (*P *= 0.006 and *P *= 0.02, respectively). Also, after the intervention, all study groups showed a higher level of SOD compared to the control group, which was statistically significant (*P *< 0.001 for all).

Consumption of synbiotic + lactic acid bread led to a significant decrease in HbA1c compared to the control bread − 0.41 ± 0.06 vs. 0.004 ± 0.02%, respectively; *P *< 0.001) and the lactic acid bread (− 0.41 ± 0.06 vs. − 0.14 ± 0.05%, respectively; *P *= 0.004). Also, synbiotic bread group showed a significant reduction in HbA1c compared to the control group (− 0.28 ± 0.06 vs. 0.004 ± 0.02%, respectively; *P *= 0.002). In addition, participants consuming synbiotic + lactic acid bread had a significant increase in serum SOD compared to those consuming lactic acid (0.87 ± 0.22 vs. − 0.54 ± 0.40 mmol/L, respectively; *P *= 0.01) or control bread (0.87 ± 0.22 vs. 0.18 ± 0.17 mmol/L, *P *= 0.02).

In the adjusted model, the findings mentioned above for HbA1c and SOD remained significant. Moreover, changes in GSH-Px were significantly higher following the consumption of synbiotic + lactic acid bread than lactic acid bread (19.02 ± 17.10 vs. − 24.05 ± 12.17 µmol/L, respectively; *P *= 0.03). Also, the consumption of synbiotic bread was accompanied by a significant reduction in serum hs-CRP compared to the lactic acid bread (− 689 ± 368.98 vs. 33.80 ± 237.60 mg/L, respectively; *P *= 0.02). However, mean changes in FPG, HOMA-IR, insulin, and TAC were not significantly different between the study groups.

## Discussion

The present clinical trial revealed that consumption of synbiotic + lactic acid bread for 8 weeks among T2D patients had favorable effects on HbA1c, plasma SOD, and GSH-Px; however, we failed to find any significant effect on FPG, serum insulin, HOMA-IR, hs-CRP, and plasma TAC.

To date, the limited studies that have investigated the effect of synbiotic foods on glucose metabolism in diabetic patients have reported contradictory findings. In an earlier study conducted by Tajadadi-Ebrahimi et al. [10], consumption of 120 g/day synbiotic bread containing Lactobacillus sporogenes (1 × 10^8^ CFU) for 8 weeks led to a significant decrease in serum insulin and HOMA-IR in T2D patients; however, it had no significant effect on FPG. Similar findings were also reported by Asemi et al. that had used beta-carotene fortified synbiotic food containing Lactobacillus sporogenes (1 × 10^7^ CFU) for 6 weeks among T2D patients [7].

In the present study, we found a significant effect of synbiotic and also, a synbiotic + lactic acid bread on HbA1C, but such a favorable effect was not observed for FPG, insulin, and HOMA-IR. This was in line with the finding of Kassaian et al. study [17], indicated that supplementation with an inulin-based synbiotic containing 4 probiotic species (1 × 10^9^ CFU for each) for 24 weeks was accompanied by a significant decrease in HbA1C among adults with pre-diabetes. Also, in a study by Shakeri et al. [18], consumption of a synbiotic bread containing Lactobacillus sporogenes (1 × 10^8^ CFU) for 8 weeks in T2D patients, did not have a significant effect on FPG. Moreover, another study showed that synbiotic supplementation containing 7 viable probiotic strains and fructo-oligosaccharide for 8 weeks in T2D patients had no significant decreasing effect on FPG, insulin, and HOMA-IR [9]. It is suggested that the efficacy of synbiotic might be varied based on the gut flora’s composition of patients since it is closely related to the lifestyle, diet, age, medication, and genetic background of each individual [19]. Therefore, differences in these factors in studies’ participants might result in the inconsistent outcomes between studies. Also, differences in the type of bacteria strain, the dosage, and the number of bacteria used might be contributed to the different results between studies.

In this study, changes in HbA1c were not accompanied by a significant improvement in FPG. It should be noted that diabetic patients that participated in our study had fairly well glycemic control. It is demonstrated that in such diabetic patients, there is a low correlation between FPG with changes in HbA1c, while postprandial glucose is better correlated. In addition, it is revealed that FPG was better correlated with HbA1c in patients with poor glycemic control [20]. Therefore, the possible reason for observing no significant improvement in FPG might be attributed to the overall glycemic control of study participants.

In this study, synbiotic + lactic acid bread significantly improved plasma SOD and GSH-Px, while it did not affect on TAC and hs-CRP, which this was in agreement with the findings of previous studies [7, 10, 21]. The exact mechanism of synbiotic on antioxidant activity has not yet been fully understood. However, it is suggested that probiotics and synbiotics could improve antioxidant activity mainly through the production of SCFAs such as butyrate. SCFAs could provide NADPH, which is necessary for GSH synthesis as well as up-regulates gene expression of interleukine-18, which has anti-inflammatory properties. Also, synbiotics could stimulate the gene expression and activity of glutamate cysteine ligase (GCL), which is involved in GSH synthesis. Other possible mechanisms that might explain the beneficial effect of synbiotics on antioxidant activity are down-regulating the genes involved in oxidative stress and toll-like receptor pathways, chelating ferrous ion, reducing the levels of free radicals, oxidized-low density lipoprotein (Ox-LDL), and 8-isoprostanes as well as suppression of pro-inflammatory cytokines and ascorbate autoxidation [22, 23]. Similarly, lactic acid Bacteria has been shown to could improve antioxidant activity mainly through removing the ferrous ion and scavenging the hydroxyl and superoxide radicals [24, 25]. Therefore, it is expected that a combination of synbiotic and lactic acid in the bread has been yielded to the favorable changes on antioxidant enzymes.

Nevertheless, in contrast to our findings, several studies had reported the beneficial effect of synbiotic supplementation on serum hs-CRP in T2D patients. Asemi et al. [9] found that synbiotic supplementation comprising multispecies probiotics and fructo-oligosaccharide for 8 weeks led to a significant decrease in serum hs-CRP. Also, in another clinical trial [8], a significant reduction in serum hs-CRP was reported following supplementation with a synbiotic product containing Lactobacillus sporogenes (1 ×10^7^ CFU) and inulin for 6 weeks. The inconsistent results reported by studies might be related to factors such as the type of probiotic strain, the number of bacteria used, the dosage of supplementation of probiotics and prebiotics, as well as the overall glycemic control of participants at baseline and the duration of intervention.

To the best of our knowledge, this is the first study to assess the effect of a bread containing synbiotic and lactic acid on glycemic status, oxidative stress, and inflammation among patients with T2D. The type of synbiotic product that we had chosen for bread preparation was a major strength of this study. *Bacillus coagulans* have been shown that could exert its probiotic benefits effectively at the high temperate and the harsh acidic and enzymatic environment of the stomach [26]. In fact, its stability at high temperature is regarded as an advantage during the baking process. Also, inulin, as a known prebiotic, has a potential role in improving the viability and growth of the various strain of probiotics especially *Bacillus coagulans* [27]. Moreover, we enriched all types of bread with beta-glucan, a soluble fiber found naturally in oats and barley. Some studies found beneficial effect of a bread enriched with beta-glucan on glycemic status in T2D patients [28, 29], which this is mediated mainly by delaying the gastric emptying and subsequently, reducing the intestinal absorption of glucose as well as stimulating the PI3K/Akt signaling pathway, which has a critical role in the regulation of glucose/insulin homeostasis [30].

However, some limitations of this study should be acknowledged. First, the study population had fairly well glycemic control at baseline, which this might be the reason for not observing the significant changes in some glycemic measures. Moreover, we did not measure the postprandial glucose to compare its changes during the intervention. This could better reflect the changes in glycaemia rather than FPG in patients with well-controlled glycemic status at baseline. Second, we assessed the inflammation based on the serum levels of hs-CRP, and because of the budget limitations, we could not measure other biomarkers of inflammation such as TNF-α and IL-6, which could be better markers of inflammation. Third, the duration of intervention was relatively short; although we could find a beneficial effect of synbiotic consumption on some biomarkers of glycemic status and oxidative stress in this short time, it is likely that a longer period might lead to the additional benefits in T2D patients. Forth, we did not evaluate the effect of baking process on the probiotic activity or the effect of added lactic acid on the rate of starch digestion in the bread, which these would be valuable in elucidating the different mechanisms of action of synbiotic and lactic acid.

## Conclusion

In conclusion, daily consumption of a synbiotic bread containing lactic acid for 8 weeks had beneficial effects on HbA1c, plasma SOD, and GSH-Px levels among T2D patients, while it did not affect FPG, serum insulin, HOMA-IR, hs-CRP, and TAC. Further studies with longer duration of follow-up are needed to confirm these findings.

## Data Availability

The datasets used during the current study are available from the corresponding author on reasonable request.

## Abbreviations

T2D: type 2 diabetes
SCFAs: short-chain fatty acids
GLP-1: glucagon-like peptide-1
IL-6: interleukin-6
TNF-α: tumor necrosis factor-α
GI: glycemic index
HbA1c: glycated hemoglobin A
FPG: fasting plasma glucose
BMI: body mass index
HOMA-IR: homeostasis model of assessment-insulin resistance
hs-CRP: high sensitivity C-reactive protein
TAC: total antioxidant capacity
SOD: superoxide dismutase
GSH-Px: glutathione peroxidase
SEM: standard error of the mean
ANCOVA: analysis of covariance
